# Trend and Co-occurrence Network of COVID-19 Symptoms From Large-Scale Social Media Data: Infoveillance Study

**DOI:** 10.2196/45419

**Published:** 2023-03-14

**Authors:** Jiageng Wu, Lumin Wang, Yining Hua, Minghui Li, Li Zhou, David W Bates, Jie Yang

**Affiliations:** 1 School of Public Health and the Second Affiliated Hospital Zhejiang University School of Medicine Hangzhou China; 2 The Key Laboratory of Intelligent Preventive Medicine of Zhejiang Province Hangzhou China; 3 Department of Biomedical Informatics Harvard Medical School Boston, MA United States; 4 Division of General Internal Medicine and Primary Care Brigham and Women’s Hospital Boston, MA United States

**Keywords:** social media, network analysis, public health, data mining, COVID-19

## Abstract

**Background:**

For an emergent pandemic, such as COVID-19, the statistics of symptoms based on hospital data may be biased or delayed due to the high proportion of asymptomatic or mild-symptom infections that are not recorded in hospitals. Meanwhile, the difficulty in accessing large-scale clinical data also limits many researchers from conducting timely research.

**Objective:**

Given the wide coverage and promptness of social media, this study aimed to present an efficient workflow to track and visualize the dynamic characteristics and co-occurrence of symptoms for the COVID-19 pandemic from large-scale and long-term social media data.

**Methods:**

This retrospective study included 471,553,966 COVID-19–related tweets from February 1, 2020, to April 30, 2022. We curated a hierarchical symptom lexicon for social media containing 10 affected organs/systems, 257 symptoms, and 1808 synonyms. The dynamic characteristics of COVID-19 symptoms over time were analyzed from the perspectives of weekly new cases, overall distribution, and temporal prevalence of reported symptoms. The symptom evolutions between virus strains (Delta and Omicron) were investigated by comparing the symptom prevalence during their dominant periods. A co-occurrence symptom network was developed and visualized to investigate inner relationships among symptoms and affected body systems.

**Results:**

This study identified 201 COVID-19 symptoms and grouped them into 10 affected body systems. There was a significant correlation between the weekly quantity of self-reported symptoms and new COVID-19 infections (Pearson correlation coefficient=0.8528; *P*<.001). We also observed a 1-week leading trend (Pearson correlation coefficient=0.8802; *P*<.001) between them. The frequency of symptoms showed dynamic changes as the pandemic progressed, from typical respiratory symptoms in the early stage to more musculoskeletal and nervous symptoms in the later stages. We identified the difference in symptoms between the Delta and Omicron periods. There were fewer severe symptoms (coma and dyspnea), more flu-like symptoms (throat pain and nasal congestion), and fewer typical COVID symptoms (anosmia and taste altered) in the Omicron period than in the Delta period (all *P*<.001). Network analysis revealed co-occurrences among symptoms and systems corresponding to specific disease progressions, including palpitations (cardiovascular) and dyspnea (respiratory), and alopecia (musculoskeletal) and impotence (reproductive).

**Conclusions:**

This study identified more and milder COVID-19 symptoms than clinical research and characterized the dynamic symptom evolution based on 400 million tweets over 27 months. The symptom network revealed potential comorbidity risk and prognostic disease progression. These findings demonstrate that the cooperation of social media and a well-designed workflow can depict a holistic picture of pandemic symptoms to complement clinical studies.

## Introduction

The global COVID-19 pandemic caused by SARS-CoV-2 has resulted in more than 630 million infections and 6.59 million deaths as of October 31, 2022 [1]. The pandemic is still ongoing, and its catastrophic impact may continue to grow and last for years. To deepen the understanding of this disease, relevant studies have been increasingly emerging, and their aims vary from determining molecular structures [2,3] to developing drugs and vaccines [4-6]. Concurrently, clinicians have endeavored to analyze clinical symptoms to guide therapeutic strategies [7]. Public health officials have also tried to investigate the prevalence of symptoms to use the findings to provide precise prevention and control strategies for both people and governments [8,9].

As a popular communication tool and public discussion platform, social media, such as Twitter, has permeated every aspect of our daily lives. Twitter has 396.5 million users globally, and at least 500 million tweets are sent daily [10]. Especially during the pandemic, social media played an essential role in information generation, dissemination, and consumption [11,12], yielding rich information about the pandemic. Therefore, there has been emerging COVID-19–related research based on big data from social media. Such studies include topics in infodemics, public attitudes, detection or prediction of confirmed cases, and government responses to the pandemic [13-15]. However, they mainly focused on thematic analysis [16,17] or sentiment analysis [18,19], and only a few studies analyzed the symptoms and their epidemic-related characteristics. For example, Huang et al [20] identified 485 related posts of COVID-19 infections seeking help on Sina Weibo in the early days of the pandemic. They found that fever was the most common symptom and ground-glass opacity was the most common pattern on chest computed tomography. Luo et al [21] applied a deep learning model that was pretrained by clinical text on tweets to extract various symptoms. Guo et al [22] extracted 36 symptoms from 30,732 tweets, including typical symptoms like sore throat, loss of taste, and loss of smell. Alanazi et al [23] and Sarker et al [24] analyzed symptom prevalence in the early stage of the pandemic based on tweets from about 200 COVID-19 users. Recently, Sarabadani et al [25] mined 58 physiological and 3 psychological symptoms from Reddit posts and analyzed their onsets and durations. Although these studies attempted to use social media for COVID-19 symptom studies, they mainly aimed at symptom identification, and commonly conducted distribution and trend analyses in the early months of the pandemic, rather than long-term and comprehensive investigations. The potential differences between self-reporting on social media and the electronic health records (EHRs) of medical institutions have been poorly investigated and discussed, although such findings may improve our understanding of the actual prevalence and evolution of symptoms in an emergent pandemic. In addition, in-depth research on interrelationships among COVID-19 symptoms and affected body parts is missing from the literature.

Current understandings of COVID-19 symptoms are primarily established on clinical data from medical institutions [26-28], such as EHRs. However, nearly 80% of patients with asymptomatic or mild-symptom infections are not promptly or never clinically diagnosed and treated [29-31], leading to potential missing information for mild and early symptoms. In addition, privacy policies on patient data have slowed cross-institutional cooperation and thorough studies of the pandemic on a large scale [32]. Due to limited data size and sample diversity, current COVID-19 symptom network analyses only include a few typical symptoms. For example, Fernández-de-Las-Peñas et al [33] included 1969 patients and conducted a network analysis with 22 symptoms to support the relevance of headache as a key onset symptom in the acute COVID-19 phase. Millar et al [34] developed a symptom network that only consists of 28 nodes to identify distinct symptom subphenotypes. It is therefore challenging to construct a holistic network of comprehensive symptoms and affected systems.

To address these research gaps, we propose an efficient workflow for tracking and analyzing the general prevalence status and relationships of COVID-19 symptoms using social media. The key contributions of this paper are as follows:

1. Develop a comprehensive hierarchical symptom lexicon that handles social media colloquialism and maps symptoms to their affected systems, including 10 affected systems, 257 symptoms, and 1808 descriptions.
2. Propose a novel workflow to investigate the symptom characteristics of an emergent pandemic using social media, including an overall analysis of quantity and distribution, a longitudinal analysis of symptom prevalence with time and virus strains, and a co-occurrence network of the pandemic symptoms and affected systems.
3. Conduct the first dynamic prevalence status and network analysis of COVID-19 symptoms using large-scale and long-term social media data, which will reveal the prevalence difference between Delta and Omicron, and construct a comprehensive symptom network to uncover the co-occurrence relationships.

## Methods

### Overall Workflow

**Figure 1:**
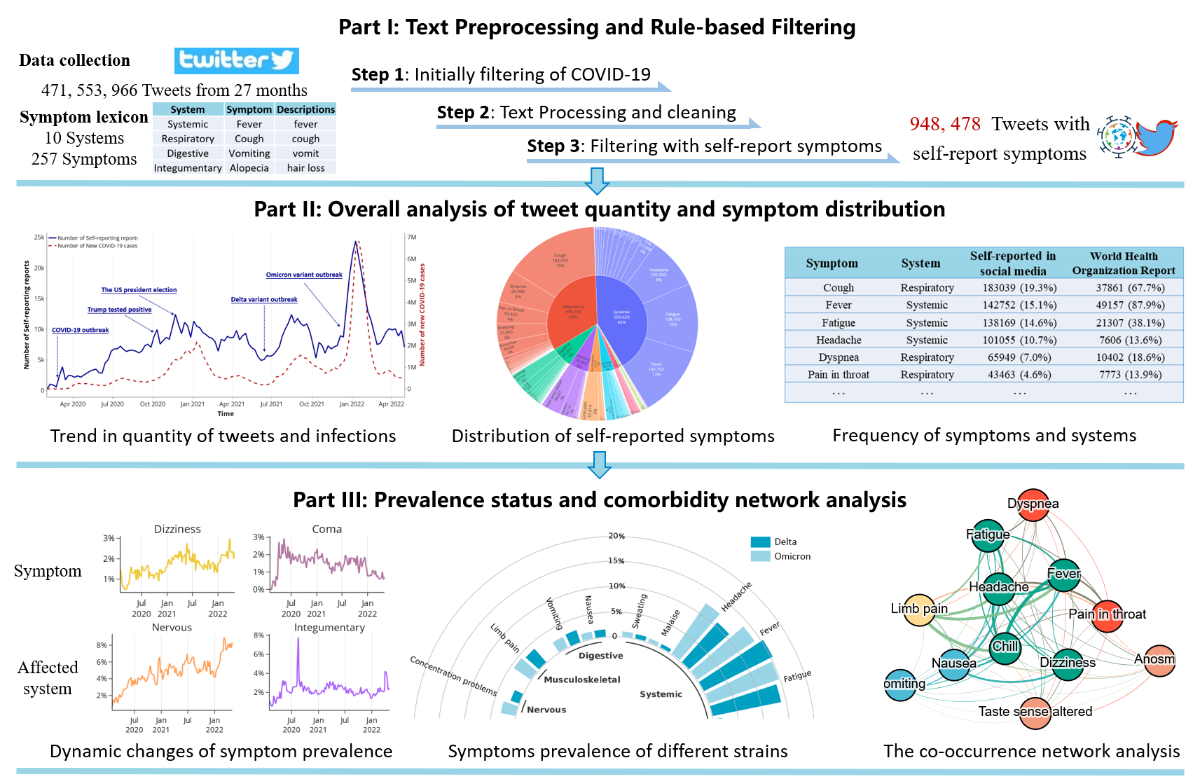
Overall workflow.

We designed a pipeline to identify and study the characteristics and co-occurrences of COVID-19 symptoms using Twitter. The overall workflow is visualized in Figure 1. It consists of 3 main parts. First, text preprocessing and rule-based filtering, which performs initial data collection, text preprocessing, and tweet filtering using a lexicon. Second, overall analysis of quantity and distribution, which conducts trend analysis on the number of COVID-19 cases and the number of tweets with self-reported symptoms. It also depicts the overall distribution and detailed frequency of identified symptoms and affected systems. Third, prevalence status and comorbidity network analysis, which further explores the dynamic evolution of symptom prevalence regarding time and virus variants and constructs a co-occurrence network to reveal in-depth relationships among symptoms and affected body systems.

### Data Collection

We selected nonretweeted English tweets related to COVID-19 using unique tweet identifiers from a widely used open-source COVID-19 tweet database [35,36]. The tweets were identified by Twitter’s trending topics and selected keywords associated with COVID-19, such as *COVID-19* and *SARS-CoV-2.* We downloaded 471,553,966 target tweets across 27 months, from February 1, 2020, to April 30, 2022, using Twitter’s application programming interface (API).

### Symptom Lexicon

Based on current literature, we built a comprehensive and hierarchical COVID-19 symptom lexicon containing synonyms of symptoms and the affected body parts [24,37-40]. The primary sources included the standard symptom corpus compiled by Wang et al [37] and Goss et al [38] based on EHRs, the COVID-19 symptom corpus compiled by Sarker et al [24], and the COVID-19 symptom keywords used by Lopez-Leon et al [39] and Mao et al [40]. Specifically, we manually checked each symptom and enriched them with colloquial variants frequently found on social media. Since Twitter users often use personalized colloquialisms rather than formal terms to describe their symptoms, the same symptom can have many referents. As a result, we considered tense, person deixis, singular/plural forms, spelling mistakes, etc, for each symptom when curating the lexicon. We used the formal names defined in the SNOMED-CT (Systematized Nomenclature of Medicine Clinical Terms) [41] and added the varied forms of the proper names as their alternative names. For example, in our lexicon, the symptom “hearing loss” (proper name in SNOMED-CT) has descriptions (mostly personalized colloquial descriptions) such as “deafness,” “difficulty hearing,” and “loss of hearing.”

In addition, we grouped symptoms according to the affected organs and systems into 10 families [42,43] as follows: cardiovascular, digestive, integumentary, musculoskeletal, nervous, reproductive, respiratory, urinary, sensory, and systemic. The final symptom lexicon contained 10 affected organs/systems, 257 symptoms, and 1808 synonyms (Multimedia Appendix 1).

### Text Preprocessing and Rule-Based Filtering

To identify tweets with self-reported symptoms for subsequent analysis, we designed a 3-step method that can be roughly summarized into filtering tweets with strict COVID-19 keywords, text cleaning, and matching of self-reported symptoms (Multimedia Appendix 2).

### Trend Analysis on the Quantity of New COVID-19–Related Tweets

We compared weekly numbers of new COVID-19 tweets to new cases in countries with the most Twitter users. A survey on Statista shows that as of January 2021 [44], the top 4 countries that have the most Twitter users and use English as their primary language are the United States, the United Kingdom, the Philippines, and Canada (Multimedia Appendix 3). We used new COVID-19 cases in these countries reported by the World Health Organization (WHO) as a rough representation of new COVID-19 cases (Multimedia Appendix 4). We calculated weekly numbers of new tweets for both before and after the filtering. We also computed their Pearson correlation coefficient with the number of new cases to examine whether there was a statistically significant association between COVID-19 severity and public response.

### Overall Distribution and Dynamic Frequency Analysis of Symptoms

Based on the COVID-19 symptom lexicon, we counted occurrences of each symptom by matching their synonyms against the filtered tweet data sets. Multiple mentions of the same symptom in one tweet were counted as one. To explore dynamic changes in symptom distribution with time, we calculated each symptom’s weekly frequency, normalized by the number of all self-reporting tweets. We also calculated the normalized frequency for each affected system.

### Comparison of the Symptom Prevalence Status Between Different Strains

COVID-19 has several variants that present different epidemic characteristics [45], such as the highly transmissible B.1.617.2 (Delta) variant [46,47] and B.1.1.529 (Omicron) variant [48], which have led to rapid global rises in cases. In this section, we compare self-reported symptom frequencies between the Delta and Omicron variants. We extracted tweets from June 1, 2021, to November 27, 2021, when Delta was the globally dominant variant [42,49,50] to represent Delta. Similarly, we extracted tweets from December 20, 2021, to April 30, 2022 [42] to represent Omicron.

We extracted symptoms from the 2 groups of tweets and selected those with ≥1% frequency as common symptoms. Then, we used the chi-square test to calculate odds ratios (ORs) for Delta versus Omicron to assess the approximate prevalence differences of these common symptoms in the 2 periods. Since a patient can get infected with Delta in the Omicron-dominated period, this method calculates the odds of detecting a symptom among infected participants during the Delta-dominated period compared to the Omicron period.

### Network Analysis

A COVID-19 patient may have multiple symptoms and report them simultaneously. Based on the symptom lexicon, we matched each symptom against each tweet to create a data set X = [*x*_1_, *x*_2_, …, *x_n_*] ϵ *R^n^*^×^*^m^*, where *x_i_* = [*d_i_*_1_, *d_i_*_2_, …, *d_im_*]. *d_ij_* is a binary feature that represents whether tweet *x_i_* mentions symptom *j*; *m* and *n* represent the numbers of symptoms and tweets, respectively.

To quantitatively explore the strength of co-occurrence between 2 symptoms, we built symptom vector V, where V = X^T^ = [*v*_1_, *v*_2_, …, *v_m_*] ϵ *R^m^*^×^*^n^*, meaning that each dimension of *v_x_* is a binary feature that indicates whether the symptom *x* was mentioned in tweet *i*. The co-occurrence strength is modeled by the similarity between the 2 symptom vectors, for which we adopted cosine similarity as the metric. In conclusion, the co-occurrence C between *v_x_* and *v_y_* can be modeled by the following equation:

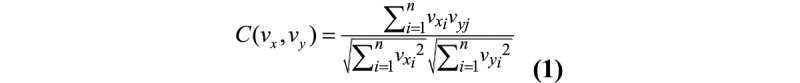



Based on the model, we constructed a weighted co-occurrence network of COVID-19 symptoms, where nodes represent symptoms and edges capture the co-occurrence strength between symptom pairs. We used Gephi [51] and ForceAtlas2 algorithm [52] to visualize the symptom network.

## Results

### Weekly Trends of Tweets With Self-Reported Symptoms

**Figure 2:**
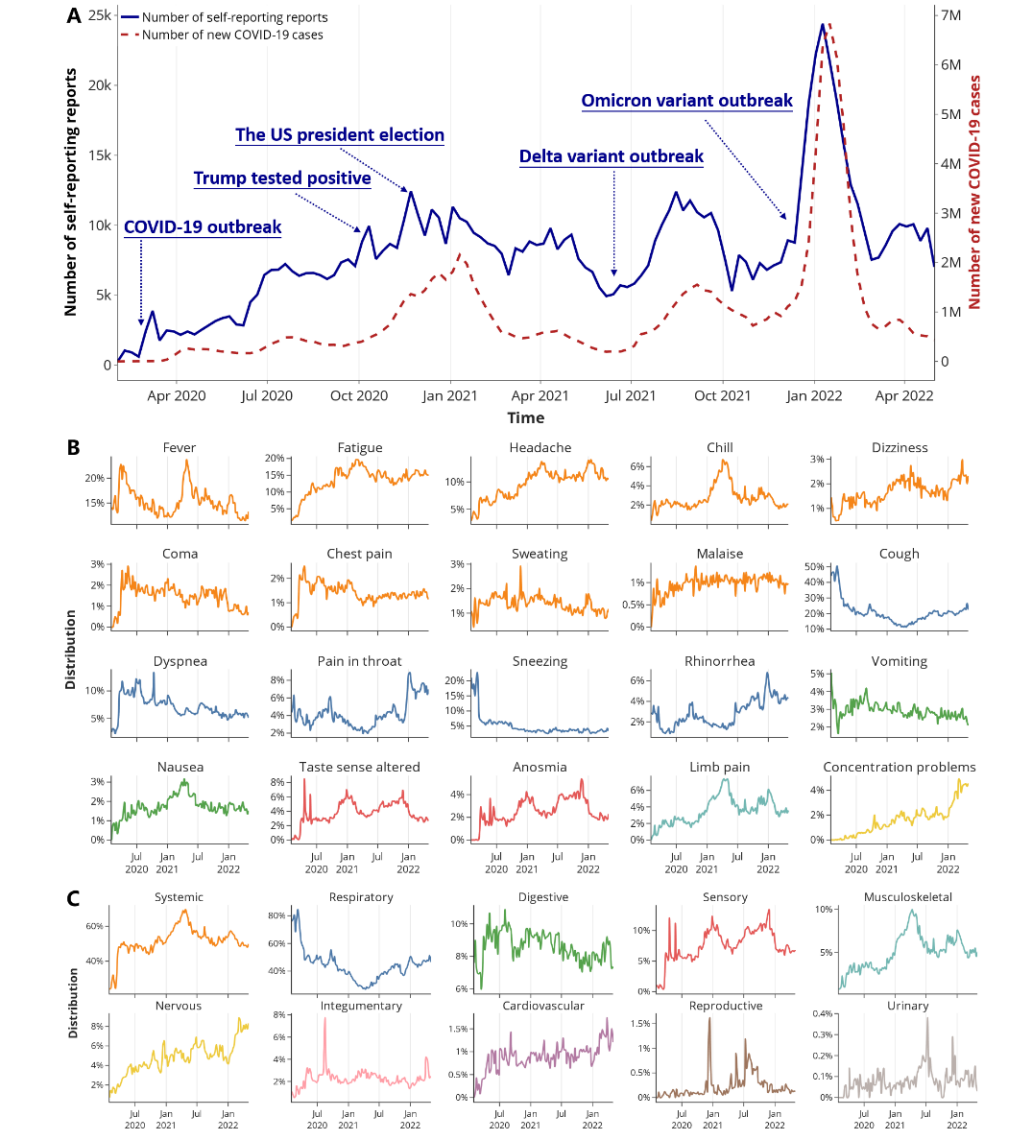
Weekly numbers of self-reporting tweets and weekly trends of the frequency of symptoms and affected systems. (A) Weekly numbers of self-reporting COVID-19 tweets and sum of new COVID-19 cases in the United States, the United Kingdom, Canada, and the Philippines. (B) Weekly trends of the frequency of the top 20 symptoms. (C) Weekly trends of the frequency of the affected systems. The colors of symptoms in (B) correspond to affected systems in (C).

We selected 948,478 unique COVID-19-related tweets with self-reported symptoms to conduct the studies. We observed that weekly changes of tweets with self-reported symptoms were roughly consistent with the trends of new cases in the 4 selected countries (Figure 2A). The Pearson correlation coefficient between the 2 trends was 0.8528 (*P*<.001) and was higher than the Pearson correlation coefficient between new cases and unfiltered COVID-19-related tweets (0.3235; *P*<.001; Multimedia Appendix 5). Moreover, self-reporting tweets showed a significant leading trend compared with new cases when the leading time was set to 1 week. Such a trend had a higher correlation (Pearson correlation coefficient=0.8802; *P*<.001) than when no time difference was set.

There were several waves of new cases and self-reporting tweets, including the initial outbreak in March 2020 and the continuous rapid spread. The first peak occurred during the transition of 2020 and 2021. Weekly new cases fell back to a prepeak level and then increased at a slow rate until the outbreak of Delta, which started a new wave of infections in the middle of 2021. Omicron swept across countries from December 2021, took over Delta, and gave rise to the most enormous COVID wave. During the week of January 16, 2022, weekly new cases reached the highest number of 6.83 million. Weekly self-reporting showed similar trends but with more fluctuations. Such fluctuations mainly happened with hotspot issues on social media. One example was when former US president Donald Trump tested positive for COVID during the presidential election.

### Distribution of COVID-19 Symptoms and Affected Organs/Systems

**Figure 3:**
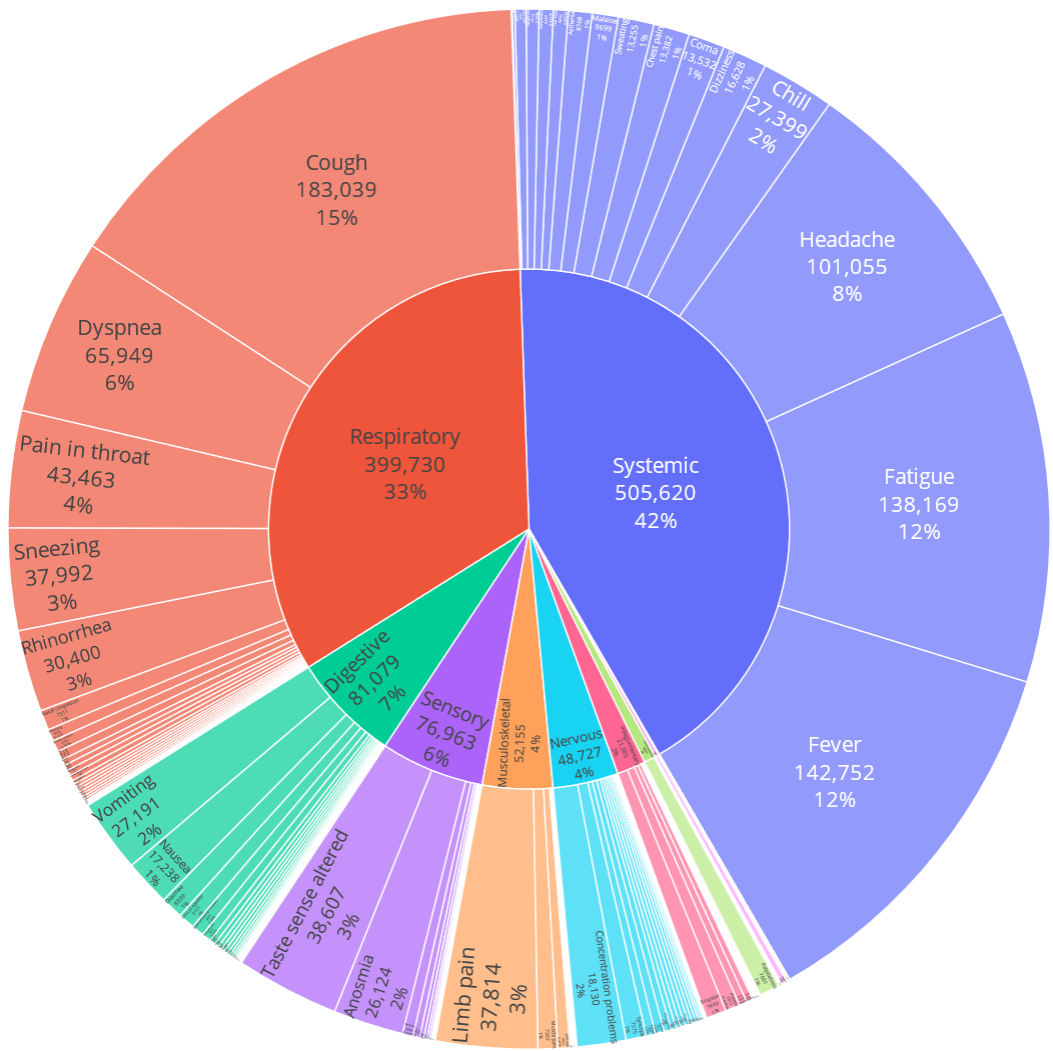
The distribution of mentioned symptoms and their affected body systems.

In all, 245 symptoms were mentioned a total of 1,197,733 times in 948,478 tweets. A total of 201 symptoms from 10 affected systems were mentioned in ≥10 tweets. The distribution of different systems and their related symptoms are hierarchically visualized in Figure 3. Notably, systemic symptoms accounted for 42.2% (505,620/1,197,733) of the total number of symptom occurrences, followed by respiratory (399,722/1,197,733, 33.4%), digestive (81,054/1,197,733, 6.8%), sensory (76,959/1,197,733, 6.4%), musculoskeletal (52,142/1,197,733, 4.4%), nervous (48,697/1,197,733, 4.1%), integumentary (21,351/1,197,733, 1.8%), cardiovascular (8839/1,197,733, 0.7%), reproductive (2418/1,197,733, 0.2%), and urinary (772/1,197,733, 0.1%) symptoms.

### Frequency of Common COVID-19 Symptoms and Affected Systems

Overall, 20 common symptoms had more than a 1% frequency (Table 1) (more details are in Multimedia Appendix 6). Note that the WHO report was based on 55,924 laboratory-confirmed cases from China in the early stage of COVID-19 [53]. The data of Delta and Omicron were extracted and calculated from our data set in the corresponding period.

Figure 2B and Figure 2C show the weekly frequency of COVID symptoms and affected systems. The frequency of symptoms showed dynamic changes with the progression of the pandemic and had some distinct waves. In the early stage of COVID-19, cough, fever, and sneezing were the major symptoms, while other symptoms were rarely reported. With the progression of the pandemic, more symptoms, such as taste sense altered, chills, and anosmia, started to emerge. Respiratory symptoms were most common initially, accounting for more than 80% of symptoms at one time and then gradually decreasing to about 40%. In contrast, the frequency of systemic, musculoskeletal, and nervous symptom mentions showed increasing trends. The frequencies of different symptoms gradually stabilized, with fluctuations associated with hotspot issues and the emergence of new variants.

**Table 1:** Occurrences and frequencies of common symptoms in filtered tweets.

Symptom	Body system	Self-reported symptoms (all; N=948,478), n (%)	WHO^a^ symptoms (N=55,924), n (%)	Self-reported symptoms (Delta) (N=149,462), n (%)	Self-reported symptoms (Omicron) (N=158,994), n (%)
Cough	Respiratory	183,039 (19.3)	37,861 (67.7)^b^	38,378 (18.4)	52,325 (21.4)
Fever	Systemic	142,752 (15.1)	49,157 (87.9)	32,501 (15.5)	34,562 (14.1)
Fatigue	Systemic	138,169 (14.6)	21,307 (38.1)	29,621 (14.2)	36,704 (15.0)
Headache	Systemic	101,055 (10.7)	7606 (13.6)	22,846 (10.9)	30,601 (12.5)
Dyspnea	Respiratory	65,949 (7.0)	10,402 (18.6)	13,841 (6.6)	13,601 (5.6)
Pain in the throat	Respiratory	43,463 (4.6)	7773 (13.9)	8381 (4.0)	18,059 (7.4)
Taste sense altered	Sensory	38,607 (4.1)	N/A^c^	10,426 (5.0)	8188 (3.3)
Sneezing	Respiratory	37,992 (4.0)	N/A	7281 (3.5)	8024 (3.3)
Limb pain	Musculoskeletal	37,814 (4.0)	8277 (14.8)^d^	8114 (3.9)	10,876 (4.4)
Rhinorrhea	Respiratory	30,400 (3.2)	2684 (4.8)^e^	7570 (3.6)	11,952 (4.9)
Chills	Systemic	27,399 (2.9)	6375 (11.4)	5890 (2.8)	5928 (2.4)
Vomiting	Digestive	27,191 (2.9)	2796 (5.0)^f^	5780 (2.8)	6408 (2.6)
Anosmia	Sensory	26,124 (2.8)	N/A	7983 (3.8)	5525 (2.3)
Concentration problems	Nervous	18,130 (1.9)	N/A	4285 (2.0)	8104 (3.3)
Nausea	Digestive	17,238 (1.8)	N/A	3675 (1.8)	4187 (1.7)
Dizziness	Systemic	16,628 (1.8)	N/A	3701 (1.8)	5047 (2.1)
Coma^g^	Systemic	13,532 (1.4)	N/A	3295 (1.6)	2028 (0.8)
Chest pain	Systemic	13,382 (1.4)	N/A	2634 (1.3)	3312 (1.4)
Sweating	Systemic	13,255 (1.4)	N/A	2511 (1.2)	3053 (1.2)
Malaise	Systemic	9699 (1.0)	N/A	2165 (1.0)	2573 (1.1)
Nasal congestion^g^	Respiratory	7511 (0.8)	N/A	1726 (0.8)	2952 (1.2)

^a^Reported by the World Health Organization (WHO) but not the top symptoms among self-reported symptoms: hemoptysis (WHO: 503, 0.9%, ranked 13th; our assessment: 614, 0.1%, ranked 75th).

^b^Specifically dry cough.

^c^N/A: not applicable.

^d^Including myalgia (limb pain) and arthralgia (joint pain).

^e^Reported as nasal congestion, including rhinorrhea and nasal congestion (count 3673, frequency 0.6%, rank 20) among self-reported symptoms.

^f^Including vomiting and nausea.

^g^For Omicron, nasal congestion reached 1.2% and replaced coma as the 20th symptom.

### Prevalence Difference in Symptoms Between COVID-19 Variants

**Figure 4:**
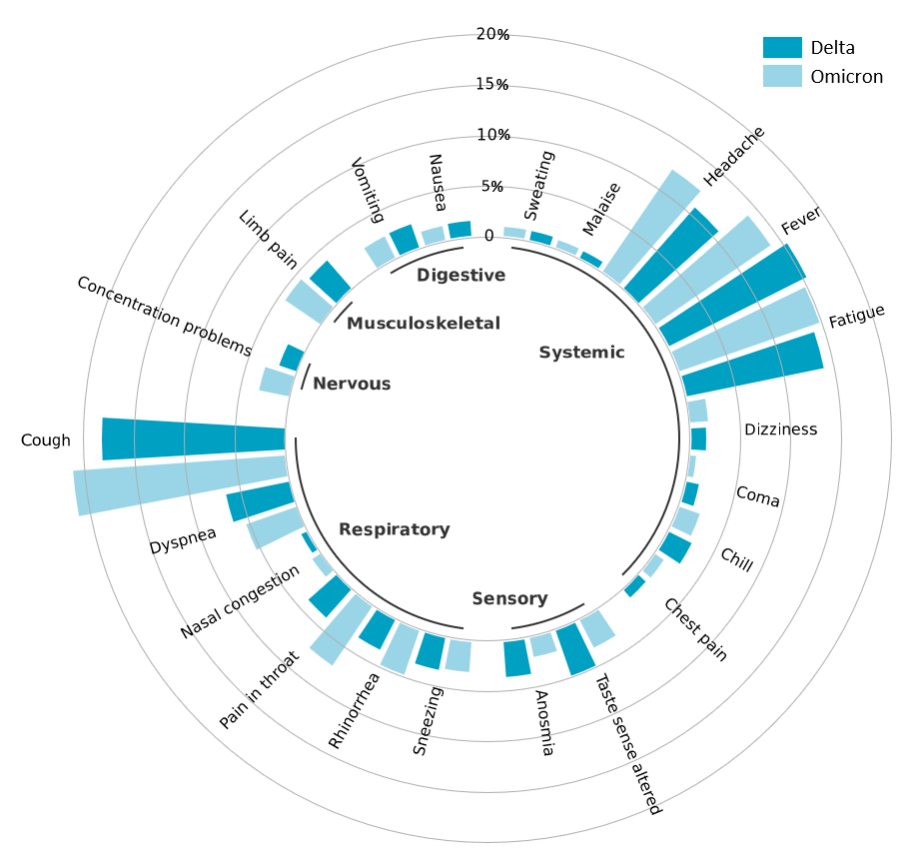
Frequency difference in common symptoms between the Delta and Omicron variants.

A total of 209,074 tweets from June 1, 2021, to November 27, 2021, were placed in the Delta group, while 244,960 tweets from December 20, 2020, to April 30, 2021, were placed in the Omicron group. Table 1 shows the top common symptoms and corresponding frequencies. Figure 4 shows the frequency differences of common symptoms for Delta versus Omicron.

The top 20 symptoms of Omicron and Delta were roughly the same, but nasal congestion replaced coma as one of the top 20 symptoms of Omicron. Among these 21 symptoms, 8 were significantly (*P*<.001) less prevalent among individuals infected during the Omicron period than during the Delta period (top 5 ORs: coma: OR 0.52, 95% CI 0.49-0.55; anosmia: OR 0.58, 95% CI 0.56-0.60; taste sense altered: OR 0.66, 95% CI 0.64-0.68; dyspnea: OR 0.83, 95% CI 0.81-0.85; chills: OR 0.86, 95% CI 0.82-0.89), and 10 were significantly more likely to occur in Omicron patients than in Delta patients (top 5 ORs: pain in the throat: OR 1.91, 95% CI 1.86-1.96; concentration problems: OR 1.64, 95% CI 1.58-1.70; nasal congestion: OR 1.47, 95% CI 1.38-1.55; rhinorrhea: OR 1.37, 95% CI 1.33-1.41; cough: OR 1.21, 95% CI 1.19-1.23). Further details are provided in Multimedia Appendix 7.

### Co-occurrence Network of COVID-19 Symptoms

**Figure 5:**
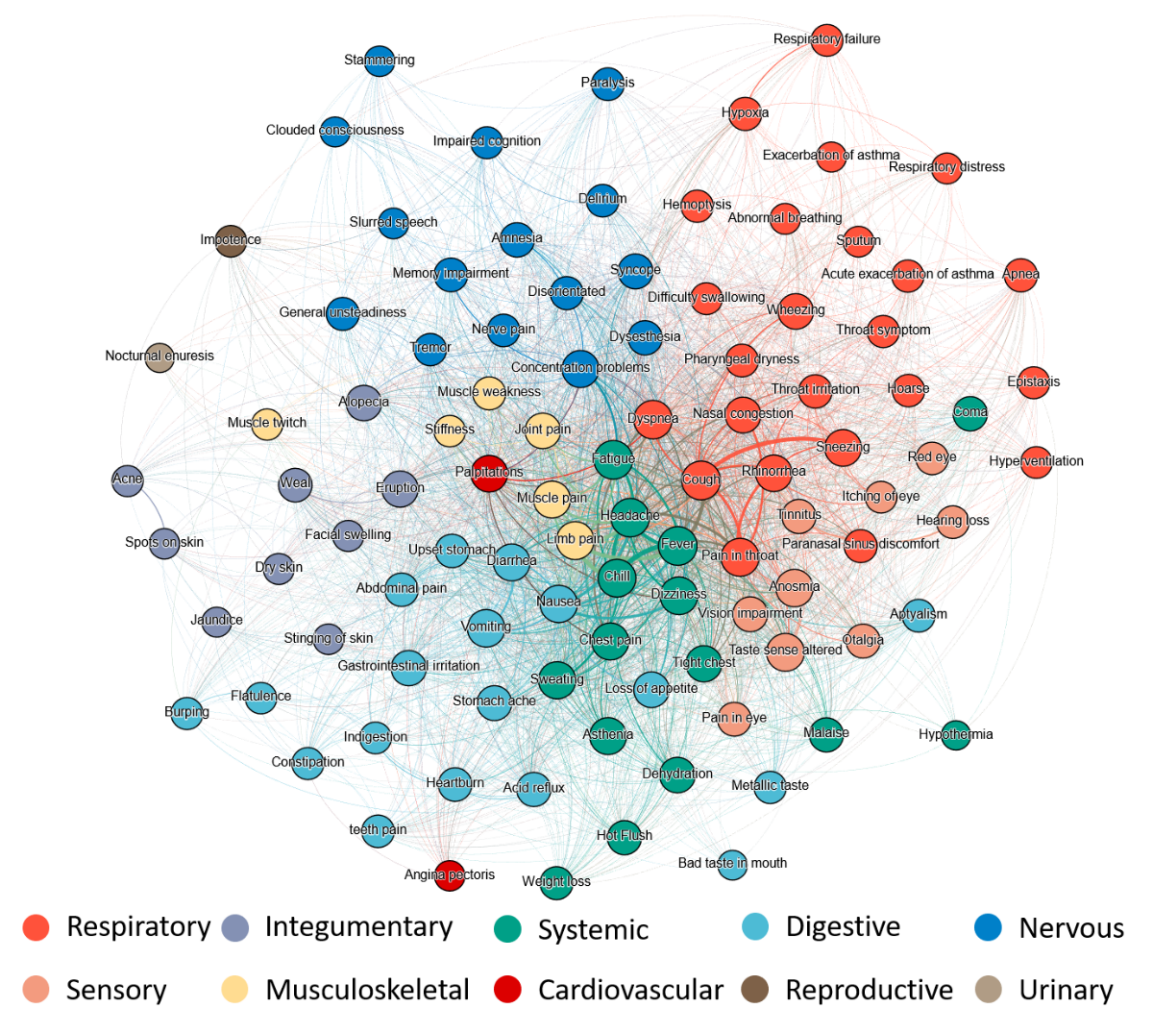
The co-occurrence network of different symptoms and affected systems.

To simplify the co-occurrence network, we selected the top 100 symptoms by their overall distribution. The final network had 100 nodes with 2654 edges (Figure 5). Overall, the symptoms in this network showed a clustering tendency according to the affected system, and the common symptoms were roughly distributed in the central region. Though systemic and musculoskeletal symptoms were not the leading part of the network, they were mainly in the center of the network and linked to the symptoms of different systems. Some outliers fell out of the clustering region of their theoretically affected systems. For example, palpitations, a cardiovascular symptom, was located at the center of the network next to systemic and musculoskeletal symptoms. Impotence, the only reproductive symptom with a high occurrence rate, and nocturnal enuresis, the only urinary symptom, were located at the network border, demonstrating that co-occurrences with other symptoms were relatively low. Both intrasystemic and intersystemic symptoms had strong co-occurrences, such as chills and fever (both systemic symptoms), palpitations (cardiovascular), and dyspnea (respiratory). For clinicians to further explore the co-occurrences of a specific symptom, we provide an interactive online version of this symptom network [54].

## Discussion

### Principal Findings

In this work, we presented a novel workflow to investigate the symptom characteristics of an emergent pandemic using social media. We curated a hierarchical symptom lexicon that handles social media colloquialism and maps symptoms to their affected systems. We constructed a comprehensive co-occurrence network for COVID-19 symptoms. To the best of our knowledge, this is the first dynamic prevalence status and network analysis of COVID-19 symptoms using large-scale and long-term social media data. This workflow can aid clinical professionals in monitoring unusual co-occurrent symptom patterns to promote pathogenesis studies. It is also promising for studying other emergent epidemics, given the accessibility and timeliness of social media.

Trend analysis on the number of tweets and COVID-19 infections demonstrated social media’s sensitivity and promptness, and emphasized its effectiveness for studying symptoms and timely monitoring pandemic status. Masri et al [55] found that new case trends could be predicted 1 week ahead based on related tweets for the 2015 Zika epidemic. In correspondence and beyond, we found a highly correlated 1-week leading trend of symptom-related tweets compared to new cases of COVID-19. This further confirms the potential of social media for predicting pandemic status. Meanwhile, small fluctuations in the trends reflected public concerns with hotspot issues such as government policies and measures regarding the pandemic. For example, Figure 2A shows that the presidential election and Trump testing positive triggered increases in self-reporting tweets. This could be attributed to people discussing relevant problems and bringing up their own experiences, including symptoms. The insights gained from this type of trend analysis could help officials better guide and warn the public during pandemics. Readers can refer to our previous study for a more detailed investigation of the influence of hotspot issues on symptom reports [56].

The common symptoms and their prevalence ranks identified in our study are mostly in accordance with WHO reports but with different frequencies. This difference may be a result of different study populations; compared to studies based on EHRs, self-reported symptoms on social media were predominantly from COVID-19 patients with mild symptoms who did not seek help from health care agencies. For example, limb pain and vomiting were relatively less common in social media than in WHO reports, and hemoptysis was reported as a common symptom in WHO reports and ranked 13th (n=503, 0.9%) in these reports, but only ranked 75th in our study (n=614, 0.1%). The gap in the prevalence ratio between social media and WHO reports may be because COVID-19 patients do not self-report all symptoms on social media. In addition, different granularity and definitions of symptoms may be related to the frequency difference. For example, cough in WHO reports only refers to dry cough, whereas wet cough is often correlated with sputum production [53]. Therefore, cough was the most common symptom in social media but was the second most common in WHO reports. Nonetheless, such strict definitions are less suitable for social media data. Using the symptom lexicon, we identified a few symptoms that were not taken seriously in the WHO’s early reports, such as taste sense altered, anosmia, and nausea [57-59]. In addition, we noticed some relatively infrequent symptoms, such as alopecia (n=5373) and impotence (n=2027). A recent large-scale study has also observed that hair loss and sexual dysfunction are typical long-COVID symptoms in nonhospitalized adults with confirmed SARS-CoV-2 infection [60]. Having learned from the UK government’s experience of being urged by general practitioners to update the official COVID-19 symptom list to eliminate confusion [61,62], policymakers should be aware that timely updates on the disease are essential to reassure the public, control the disease, and better manage patients with specific complications.

The longitudinal analysis of symptom prevalence showed that COVID-19 is a multiorgan disease with broad-spectrum manifestations and that its symptom prevalence dynamically varies over time. As the key receptors of SARS-CoV-2 are highly co-expressive in the respiratory tract [63-65], the initial symptoms are mainly respiratory and systemic symptoms caused by inflammation. However, over time, extensive self-reports of multiple symptoms from different systems confirmed that COVID-19 is a multiorgan disease [66]. At the later stage of the pandemic, there are increasing reports of persistent symptoms after COVID-19, such as fatigue, concentration problems, and limb pain (muscle/joint) [67,68]. Notably, consistent with recent findings on the increased risks of cardiovascular diseases [69] and long neuropsychiatric symptoms [70], our results showed a burst of attention toward nervous and cardiovascular symptoms on social media in January 2022, which has continued growing. This alerts us to the emerging prolonged signs (long COVID) [71] and their chronic burden on the nervous and cardiovascular systems.

The comparison of symptom prevalence between Delta and Omicron demonstrated that our method can promptly seize the epidemic characteristics and common symptom spectrum of new viruses. As reported by the general population, Omicron has (1) lower ORs for severe symptoms, such as coma and dyspnea; (2) higher ORs for flu-like symptoms, such as pain in the throat, concentration problems, nasal congestion, and rhinorrhea; and (3) lower ORs for some typical COVID symptoms, such as anosmia and taste sense altered [42,72]. This finding confirms that the Omicron variant is much more transmissible than previous variants but has less severe symptoms [73,74].

The network of COVID symptoms and affected systems, built on massive data and a comprehensive lexicon, contains more extensive information than previous studies [33,34]. While symptoms of the same system have higher co-occurrences, we did observe intersystem co-occurrences consistent with clinical studies. For example, coma exhibited strong relationships with respiratory symptoms in our networks, especially dyspnea, because the hypoxic/metabolic changes caused by an intense inflammatory response can trigger a cytokine storm and may further result in coma and encephalopathy [75]. We also found unusual co-occurrences. For example, palpitations as a cardiovascular symptom strongly correlated with dyspnea and dizziness (respiratory and systemic) [76]. Impotence, a reproductive symptom, had the strongest correlation with alopecia (an integumentary symptom). They both showed higher hazard ratios in people who experienced long COVID [60], and they may be related to the high expression of key receptors of SARS-CoV-2 (ACE2 [angiotensin-converting enzyme 2] and TMPRSS2 [transmembrane protease, serine 2] [77,78]) in reproductive organs and the androgen-mediated SARS-CoV-2 infection [79]. Recent studies [77,80,81] found that men with male pattern hair loss (caused by elevated androgen signaling) were at a higher risk of experiencing more severe COVID-19 symptoms. Furthermore, many studies adopted antiandrogens as a clinical treatment option for COVID-19 [77]. Although the exact mechanism requires more rigorous studies, these strong relationships among unexpected groups of symptoms may point to new foci of disease progression or indicate the potential risk of co-occurrent symptoms.

Urgent pandemics and outbreaks, such as COVID-19 and the recent monkeypox outbreak, always attract considerable discussions on social media [82]. These discussions contain rich information about the pandemic. Big data on social media can mitigate potential information gaps in hospital-based epidemiologic studies when many patients are not timely diagnosed and treated. Moreover, the promptness of social media supports a fast-track symptom spectrum and dynamic changes in symptom prevalence, providing clues to enlighten clinical treatment and pathogenesis investigations. These advantages in terms of efficiency and availability make our workflow promising for monitoring and analyzing emergent pandemics.

### Limitations

We acknowledge that our study has limitations. First, although we reviewed substantial studies to construct a lexicon that is as comprehensive as possible, it would have inevitably missed some colloquial variants of symptoms due to the noisy nature of Twitter. Second, the self-reported symptoms and cases were not laboratory-confirmed results. Moreover, some of our analyses could be biased. For example, we split the dominant period of different strains based on reports from the WHO and Centers for Disease Control and Prevention of the United Kingdom and the United States, but patients infected in one period still had chances of infection from another strain. Therefore, we explicitly point out that our comparison is an estimation. Third, due to the desultory and noisy nature of social media data, users usually did not report precisely the infection timeline and symptom duration, so we could not accurately distinguish the tweets regarding long COVID and initial infection. We manually checked the sampled tweets (n=200) to extract the descriptions of symptoms and the timeline. Only 5% were explicitly related to long COVID. Therefore, we did not analyze these tweets separately. The huge volume of social media data has alleviated such an impact, and the finding still reflected the actual prevalence status, which was also consistent with previous reports and studies. Finally, like every other public health study based on social media, our study has potential cohort bias as the demographic distribution of social media does not represent that of the whole population.

### Conclusions

We developed a novel workflow to explore the dynamic characteristics of pandemic symptoms through social media. Using symptom analysis, we performed a large-scale and long-term social media–based study on COVID-19 and identified 201 symptoms from 10 systems. Compared to clinical data–based studies, we found a different symptom prevalence reported by a population of predominantly mild-symptom patients. Evaluations of the big data of social media can complement clinical studies to depict a more holistic picture of COVID-19 symptoms. The network revealed unusual co-occurrent symptom patterns, which may enable downstream pathogenesis studies. Owing to the accessibility and timeliness of social media, this workflow is also promising for contributing to future public health studies, such as those involving other emergent epidemics.

## Appendix

Multimedia Appendix 1 Symptom lexicon.

Multimedia Appendix 2 Pipeline of text preprocessing and rule-based filtering.

Multimedia Appendix 3 Leading 20 countries that use Twitter as of January 2021.

Multimedia Appendix 4 Weekly trends of the numbers of tweets with COVID-19 symptoms and new COVID-19 cases.

Multimedia Appendix 5 Weekly numbers of COVID-19–related tweets and new COVID-19 cases in the United States, the United Kingdom, Canada, and the Philippines.

Multimedia Appendix 6 Symptom frequency.

Multimedia Appendix 7 The different odds ratios of common systems between the original variant and the Delta variant.

## Abbreviations

EHR: electronic health record
OR: odds ratio
SNOMED-CT: Systematized Nomenclature of Medicine Clinical Terms
WHO: World Health Organization

## Data Availability

The code and Twitter IDs of this study are available at https://github.com/Dragon-Wu/COVID19-Symptoms-Twitter. Other data that support the findings of this study are available upon reasonable request.
