# Supplementary material for: Trend and Co-occurrence Network of COVID-19 Symptoms From Large-Scale Social Media Data: Infoveillance Study
Source: J Med Internet Res. 2023 Mar 14;25:e45419. doi: 10.2196/45419 (PMC10131634; doi:10.2196/45419)
Supplement: Multimedia Appendix 2 [file jmir_v25i1e45419_app2.docx]

**Multimedia Appendix 2.** Pipeline of text preprocessing and rule-based filtering.

1. Initial filtering of COVID-19

The original keywords used to collect the COVID-19-related Tweets by Chen et al. [35] and Lopez et al. [36] were not strict enough, such as *China*, *Wuhan*, and *N95*. The tweets collected by these broad keywords were mainly about the pandemic outbreak rather than self-reporting about COVID-19. To improve the efficiency of subsequent analyses, we re-filtered the original dataset by matching tweets with at least one mention of keywords directly associated with COVID-19 (Table S1). To filter negated tweets (e.g., “… don’t have COVID-19 …”), we removed tweets containing negative indicators around COVID-19-related keywords. For example, tweets with *non-* before the keywords, and tweets with mentions of *no*, *ot*, *n't*, *nor*, etc. (Table S1) within 5 words around the keywords.

1. Processing and cleaning

To ensure that the tweets contain self-reported symptoms, we removed tweets with URLs since most of such tweets are retweets. To facilitate subsequent extraction of symptoms and analysis, we removed the non-English words and non-text contents, such as line breaks, extra spaces, emojis, and mentions of usernames. Notably, many phrases contain symptom-related keywords but do not mean symptoms, such as the symptom *tired* in the phrase *tired of* and the symptom *flush* in *flush the toilet.* Therefore, we applied regular expressions to mask such misleading patterns (Table S1). Finally, we removed duplicated tweets based on the first 50 characters.

1. Filtering with self-report symptoms

First-person-related keywords (i.e., I, I’m, me, my) and symptom descriptions from the lexicon were used to case-insensitively match against the filtered tweets.

**Table S1.** Keywords and regular expressions for text preprocessing and rule-based filtering

| **Types** | **Examples** | |
| --- | --- | --- |
| Strongly relevant to COVID-19 | | Coronavirus \| corona virus \| Koronavirus \| Corona \| Ncov \| sars-cov-2 \| sars cov 2 \| sarscov \| Wuhancoronavirus \| chinese virus \| chinesevirus \| china virus \| chinavirus \| covid \| Kungflu \| kung flu \| quarentinelife \| stayhome \| Epidemic \| pandemic \| pandemie \| trumppandemic \| trump pandemic \| deltavariant \| omicron \| omicronvariant |
| Negative words | | no \| not \| n't \| n’t \| never \| none \| neither \| nor \| without \| lack \| in the absence of \| instead of \| exclusive of \| short of \| rather than \| hardly \| scarcely \| barely \| seldom |
| Keywords of first-person | | I \| I’m \| I am \| me \| my |
| Patterns for phrases that not mean symptoms | | tired of \| faint of \| flush toilet \| fake cough \| in bad taste \| a bad memory \| (((cough(\|ed\|ing\|s))\|(sneez(e\|ed\|es\|ing\|y))) (cause\|cuz\|because\|bc\|in\|over\|on\|works) ) \| ((someone\|always\|could\|every\|fake\|fakes\|faked) ((cough(\|ed\|ing\|s))\|(sneez(e\|ed\|es\|ing\|y)))( \|,\|\.)) \| ((tired )\|((to\|really\|actually\|already\|so\|don\|look\|this\|are\|were\|'re\|'s\|was\|is\|your\|our\|her\|his) tired(,\|\.))) \| ((a\|the\|is very\|a bit\|some\|is\|fake\|faked\|fakes) (faint(\|ed\|ing))( \|,\|\.)) |
