# Supplementary material for: Trend and Co-occurrence Network of COVID-19 Symptoms From Large-Scale Social Media Data: Infoveillance Study
Source: J Med Internet Res. 2023 Mar 14;25:e45419. doi: 10.2196/45419 (PMC10131634; doi:10.2196/45419)
Supplement: Multimedia Appendix 3 [file jmir_v25i1e45419_app3.docx]

**Multimedia Appendix 3.** Leading 20 countries that use Twitter as of January 2021.

| **Country** | **Twitter Users (in millions)** | | **English is the Official**  **Language** | | **English is the Primary**  **Language** |
| --- | --- | --- | --- | --- | --- |
| United States | | 69.3 | | Yes | Yes |
| Japan | | 20.9 | | No | - |
| India | | 17.5 | | Yes | No |
| United Kingdom | | 16.45 | | Yes | Yes |
| Brazil | | 16.2 | | No | - |
| Indonesia | | 14.05 | | No | - |
| Turkey | | 13.6 | | No | - |
| Saudi Arabia | | 12.45 | | No | - |
| Mexico | | 11 | | No | - |
| France | | 8 | | No | - |
| Philippines | | 7.85 | | Yes | Yes |
| Spain | | 7.5 | | No | - |
| Thailand | | 7.35 | | No | - |
| Canada | | 6.45 | | Yes | Yes |
| Germany | | 5.8 | | No | - |
| South Korea | | 5.15 | | No | - |
| Argentina | | 5 | | No | - |
| Egypt | | 3.7 | | No | - |
| Colombia | | 3.35 | | No | - |
| Malaysia | | 3.35 | | Yes | No |
| Total | | 254.95 | | - | - |
