# Supplementary material for: Trend and Co-occurrence Network of COVID-19 Symptoms From Large-Scale Social Media Data: Infoveillance Study
Source: J Med Internet Res. 2023 Mar 14;25:e45419. doi: 10.2196/45419 (PMC10131634; doi:10.2196/45419)
Supplement: Multimedia Appendix 5 [file jmir_v25i1e45419_app5.docx]

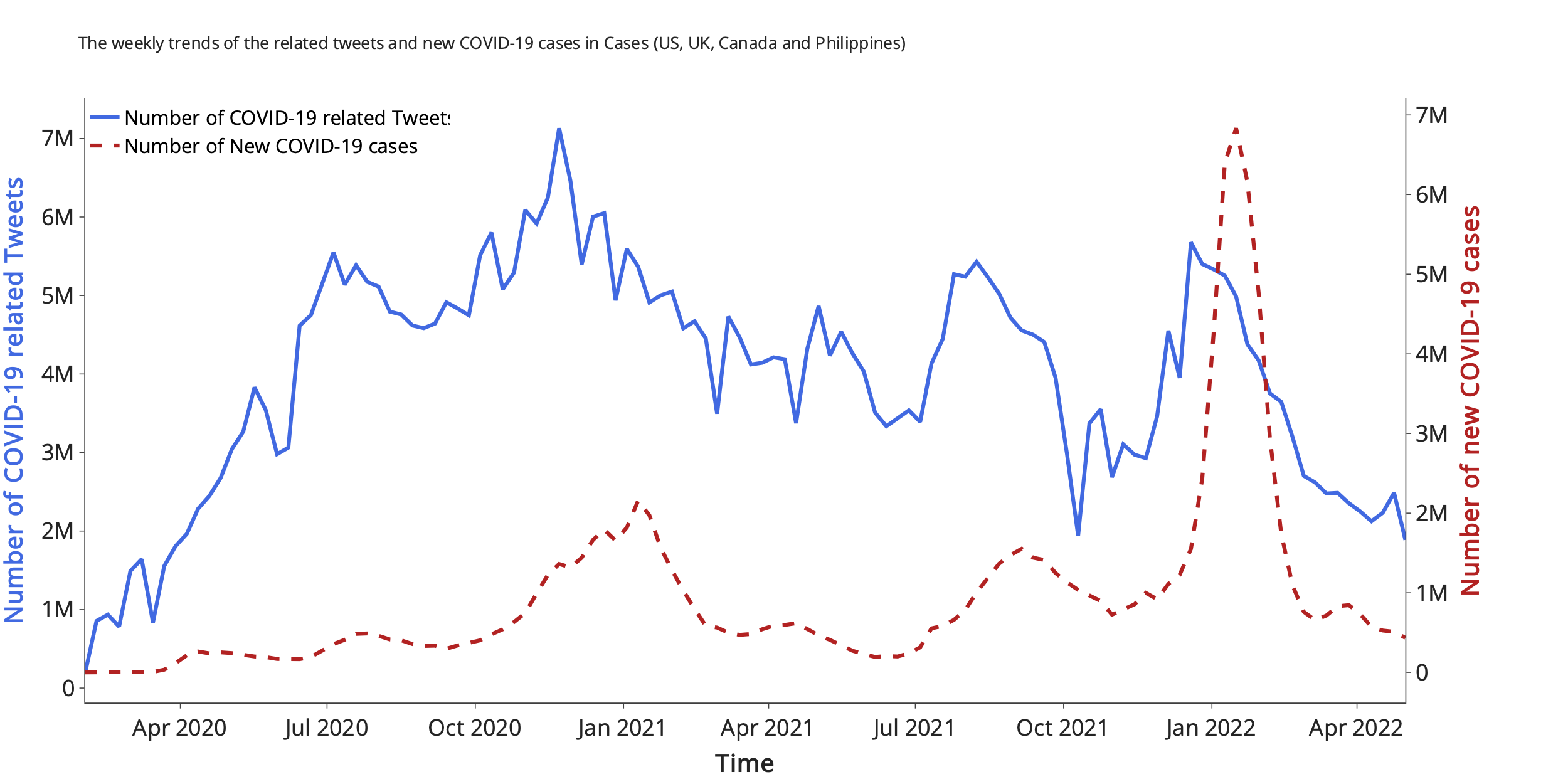


**Multimedia Appendix 5.** Weekly numbers of COVID-19–related tweets and new COVID-19 cases in the United States, the United Kingdom, Canada, and the Philippines.
