# Supplementary material for: Trend and Co-occurrence Network of COVID-19 Symptoms From Large-Scale Social Media Data: Infoveillance Study
Source: J Med Internet Res. 2023 Mar 14;25:e45419. doi: 10.2196/45419 (PMC10131634; doi:10.2196/45419)
Supplement: Multimedia Appendix 7 [file jmir_v25i1e45419_app7.docx]

**Multimedia Appendix 7.** The different odds ratios of common systems between the original variant and the Delta variant.

| **SNOMED** | **OR** | **P** | **95% CI** | **Chi2** | **Count and prevalence of Delta** | **Count and prevalence of Omicron** |
| --- | --- | --- | --- | --- | --- | --- |
| Coma | 0.5213 | <0.01 | [0.49 0.55] | 544.8633 | 3295 (1.6%) | 2028 (0.8%) |
| Anosmia | 0.5813 | <0.01 | [0.56 0.60] | 954.3881 | 7983 (3.8%) | 5525 (2.3%) |
| Taste sense altered | 0.6589 | <0.01 | [0.64 0.68] | 775.579 | 10426 (5.0%) | 8188 (3.3%) |
| Dyspnea | 0.8292 | <0.01 | [0.81 0.85] | 226.4865 | 13841 (6.6%) | 13601 (5.6%) |
| Chill | 0.8555 | <0.01 | [0.82 0.89] | 70.1969 | 5890 (2.8%) | 5928 (2.4%) |
| Fever | 0.8925 | <0.01 | [0.88 0.91] | 184.7625 | 32501 (15.5%) | 34562 (14.1%) |
| Sneezing | 0.9386 | <0.01 | [0.91 0.97] | 14.8189 | 7281 (3.5%) | 8024 (3.3%) |
| Vomiting | 0.9448 | <0.01 | [0.91 0.98] | 9.5393 | 5780 (2.8%) | 6408 (2.6%) |
| Nausea | 0.9719 | 0.2118 | [0.93 1.02] | 1.5588 | 3675 (1.8%) | 4187 (1.7%) |
| Malaise | 1.0145 | 0.6234 | [0.96 1.07] | 0.2411 | 2165 (1.0%) | 2573 (1.1%) |
| Sweating | 1.0382 | 0.1666 | [0.98 1.09] | 1.9136 | 2511 (1.2%) | 3053 (1.2%) |
| Fatigue | 1.0677 | <0.01 | [1.05 1.09] | 60.2058 | 29621 (14.2%) | 36704 (15.0%) |
| Chest pain | 1.0742 | <0.01 | [1.02 1.13] | 7.4219 | 2634 (1.3%) | 3312 (1.4%) |
| Limb pain | 1.1507 | <0.01 | [1.12 1.18] | 87.9483 | 8114 (3.9%) | 10876 (4.4%) |
| Headache | 1.1637 | <0.01 | [1.14 1.19] | 266.0105 | 22846 (10.9%) | 30601 (12.5%) |
| Dizziness | 1.1674 | <0.01 | [1.12 1.22] | 50.2551 | 3701 (1.8%) | 5047 (2.1%) |
| Cough | 1.2081 | <0.01 | [1.19 1.23] | 636.9268 | 38378 (18.4%) | 52325 (21.4%) |
| Rhinorrhea | 1.3654 | <0.01 | [1.33 1.41] | 434.1302 | 7570 (3.6%) | 11952 (4.9%) |
| Nasal congestion | 1.4654 | <0.01 | [1.38 1.55] | 159.3566 | 1726 (0.8%) | 2952 (1.2%) |
| Concentration problems | 1.6352 | <0.01 | [1.58 1.7] | 673.4027 | 4285 (2.0%) | 8104 (3.3%) |
| Pain in throat | 1.9059 | <0.01 | [1.86 1.96] | 2327.0089 | 8381 (4.0%) | 18059 (7.4%) |
